# Longitudinal Tracking of Human Fetal Cells Labeled with Super Paramagnetic Iron Oxide Nanoparticles in the Brain of Mice with Motor Neuron Disease

**DOI:** 10.1371/journal.pone.0032326

**Published:** 2012-02-27

**Authors:** Paolo Bigini, Valentina Diana, Sara Barbera, Elena Fumagalli, Edoardo Micotti, Leopoldo Sitia, Alessandra Paladini, Cinzia Bisighini, Laura De Grada, Laura Coloca, Laura Colombo, Pina Manca, Patrizia Bossolasco, Francesca Malvestiti, Fabio Fiordaliso, Gianluigi Forloni, Massimo Morbidelli, Mario Salmona, Daniela Giardino, Tiziana Mennini, Davide Moscatelli, Vincenzo Silani, Lidia Cova

**Affiliations:** 1 Mario Negri Institute for Pharmacological Research, Milan, Italy; 2 Department of Neurology and Laboratory of Neuroscience, IRCCS Istituto Auxologico Italiano, Cusano Milanino, Italy; 3 Laboratorio di Citogenetica, ISCCR Istituto Auxologico Italiano, Cusano Milanino, Italy; 4 Dipartimento di Farmacologia, Chemioterapia e Tossicologia Medica, Fondazione Matarelli, Università degli Studi di Milano, Milan, Italy; 5 Institute for Chemical and Bioengineering, ETH Zurich, Zurich, Switzerland; 6 Dipartimento Chimica Materiali e Ingegneria Chimica G. Natta, Politecnico di Milano, Milano, Italy; 7 Department of Neurology and Laboratory of Neuroscience, Centro “Dino Ferrari”, IRCCS Istituto Auxologico Italiano, Università degli Studi di Milano, Milan, Italy; University of Edinburgh, United Kingdom

## Abstract

Stem Cell (SC) therapy is one of the most promising approaches for the treatment of Amyotrophic Lateral Sclerosis (ALS). Here we employed Super Paramagnetic Iron Oxide nanoparticles (SPIOn) and Hoechst 33258 to track human Amniotic Fluid Cells (hAFCs) after transplantation in the lateral ventricles of wobbler (a murine model of ALS) and healthy mice. By *in vitro*, *in vivo* and *ex vivo* approaches we found that: 1) the main physical parameters of SPIOn were maintained over time; 2) hAFCs efficiently internalized SPIOn into the cytoplasm while Hoechst 33258 labeled nuclei; 3) SPIOn internalization did not alter survival, cell cycle, proliferation, metabolism and phenotype of hAFCs; 4) after transplantation hAFCs rapidly spread to the whole ventricular system, but did not migrate into the brain parenchyma; 5) hAFCs survived for a long time in the ventricles of both wobbler and healthy mice; 6) the transplantation of double-labeled hAFCs did not influence mice survival.

## Introduction

The evidence that Amyotrophic Lateral Sclerosis (ALS) also involves areas apart from the motor system supports the idea of a multisystemic disease, affecting multiple cell types, which requires therapeutic treatments able to provide an healthy environment for degenerating motor neurons and also capable of enhancing endogenous repair [1]. The potential of Stem Cell (SC) therapy has been widely demonstrated in different pre-clinical models of ALS [2] with a significant delay in neurological progression deriving more from the ability of SCs to produce and release several neuroprotective factors (bystander effect) than a direct replacement of degenerating neurons [3], [4]. Nevertheless, no conclusive data on the optimal SC source or delivery route are currently available either in animal models [5] or in patients [1].

We have recently published a paper describing the therapeutic efficacy of human cord blood mononuclear cells, labeled with Hoechst 33258, in two murine models of ALS by direct administration into brain ventricles [6]. Similarly to intravenous delivery [7], no migration towards the spinal cord was observed, thus confirming that the beneficial role of transplanted cells is independent from their permanence in the site of administration and their distribution in degenerating host tissues.

The wobbler mouse, a model of spontaneous motor degeneration, is characterized by selective motor neuron death affecting the cervical spinal cord region with no evident features of degeneration in upper motor neurons of motor cortex [8], [9], [10]. The wobbler pathology is not exclusively confined to the cervical spinal cord, but spreads to bulbar motor neurons and neurons from cerebellum and thalamus [11]. In addition, a significant reduction of N-acetylaspartate, a putative neuronal marker, in whole wobbler mice brains has been reported by proton magnetic resonance spectroscopy [12]. In spite of the encouraging cell grafting results in murine models of ALS, a careful investigation into the interaction between transplanted cells and host tissues is an interesting missing part of preclinical studies. In this context, several SC labeling strategies have been proposed. Among them, Super Paramagnetic Iron Oxides nanoparticles (SPIOn) appeared particularly promising for tracking different types of SCs [13].

In the present study, SPIOn were coupled with the nuclear vital dye Hoechst 33258 to label a promising source of multipotent SCs, Amniotic Fluid Cells (hAFCs). hAFCs are an heterogeneous population, routinely cultured for genetic prenatal diagnostic testing, containing several undifferentiated and committed progenitor cells whose potential has not been extensively investigated. Therefore, they constitute an ethically acceptable alternative to embryonic SCs, maintaining, at the same time, a comparable multipotentiality and a very low immunogenic response. The finding that hAFCs are able to express and release numerous cytokines and neuro-glial factors [14] further encouraged their application in the field of motor neurodegenerative disorders.

To exclude the possibility that our dual labeling strategy might lead to significant cell toxicity, the most important parameters of cell biology were evaluated before hAFC transplantation into the lateral ventricles of healthy and wobbler mice. Our hypothesis is that SC administration to the lateral ventricles could favor their distribution throughout the pathological environment of wobbler mice by cell flow to different Central Nervous System (CNS) areas.

Our *in vivo* studies focused exclusively on hAFC tracking over time in different brain regions before approaching future studies on possible therapeutic benefits of hAFCs in wobbler mice.

The results emerging from the present work provide interesting suggestions for future applications of this strategy and SC type to (pre)clinical therapy.

## Materials and Methods

### *In vitro* measurements

Dynamic Laser Light Scattering (DLLS) (Malvern Instruments, Malvern, UK) was used weekly to measure size, dispersity and z-potential of carboxydextran coated SPIOn (Endorem®; AMI-25; 11.2 mg Fe/ml, Guerbet, Roissy, France) incubated in distilled water, saline solution and amniocyte medium (Amniomax II, Invitrogen, Carlsbad, CA, USA).

SPIOn visualization was performed with an Atomic Force Microscope (AFM) coupled to NanoScope V system (Veeco instruments, Plainview, NY USA) operating in tapping mode as previously described [15].

Electron Spectroscopic Imaging (ESI) was employed to show the distribution of iron domains in SPIOn using a three-window method with an Energy Filter Transmission Electron Microscope (EFTEM, Zeiss LIBRA® 120 Oberlokochen, Germany) for the overlapping of images with electrons of iron-specific energy loss to reference images after the two background windows were subtracted.

### Amniotic fluid cell collection and culture

hAFCs were obtained from amniotic fluid samples collected after written informed consent (approved by the Ethical Committee of IRCCS-Istituto Auxologico Italiano) from pregnant women between the 16^th^ and 21^st^ week of gestation. Cells were isolated and grown for one week in Amniomax II (Invitrogen) at 37°C and 5% CO_2_ in a fully humidified atmosphere. Standard culture procedures and the Q-banding of chromosomes using quinacrine (QFQ banding technique) were used for the fetal karyotype study [16], [17]. After cytogenetic analysis, exceeding cultures from pregnancies with a normal fetal karyotype were utilized for research purposes, as specified in the informed consent. In the present work, we used four pools of hAFCs, each one obtained from 4 different fetal donors (2 with a 46,XX and 2 with a 46,XY karyotype, respectively; 15^th^–17^th^ gestational week). This approach was chosen in order to overcome any possible age/donor/sex-related variation as well as to ensure comparable stocks were used for the subsequent graft procedures. Cells obtained from each donor were harvested after trypsinization, centrifuged at 1500×g for 10 minutes, pooled together and counted. The cellular supernatants were collected as well, to avoid the loss of dividing cells in suspension. Pools were then frozen in aliquots (560,000 cells/aliquot) and stored in liquid nitrogen (ready to use). To check if enzymatic treatments and subcultures altered hAFC chromosome constitution, karyotype was periodically monitored through routine cytogenetic analysis, as described above.

### Tracer internalization and staining procedures

For internalization experiments, hAFCs were maintained for 72 hours in Amniomax II containing SPIOn (35 µg/ml), previously incubated with Poly-L-Lysine (PLL, Sigma Aldrich, St. Louis, MO, USA), as described by Bulte and colleagues [18].

After SPIOn incubation, the nuclear dye Hoechst 33258 (2 µg/ml, Sigma Aldrich) was added to hAFCs for 3 hours [6].

A diagnostic kit for the detection of ferric iron (PERLS, BioOptica, Milan, Italy) was employed to stain hAFCs following the manufacturer's instructions. Images were captured by a CCD camera connected to a Leica DMRS/HCS (Wetzlar, Germany) microscope.

hAFCs were immunostained with a specific primary antibody against the carboxydextran (1∶1,000, StemCell Technologies, Vancouver, Canada) followed by a goat-anti mouse secondary antibody (1∶1,000, Alexa-546, Molecular Probes, Invitrogen) in PBS with Normal Goat Serum (NGS). The fluorescence was visualized by Olympus Fluoview microscope BX61 (Tokyo, Japan) with confocal system FV500 equipped by specific lasers (λexc = 405 nm and λexc = 546 nm) to visualize Hoechst 33258 and Alexa-546 respectively. To avoid possible false positive results, immunofluorescence experiments were also done using the primary or the secondary antibodies alone without observing any staining.

### Transmission Electron Microscopy (TEM)

hAFCs were pre-fixed with 4% paraformaldehyde and 1% glutaraldehyde in Hepes 0.2 M (pH 7.4) and fixed at 4°C in 1% glutaraldehyde in Hepes 0.2 M, pH 7.4 until use.

Thirty minutes after incubation with 1% osmium, hAFCs were incubated for 5 minutes at Room Temperature (RT) with a saturated solution of thiocarbohydrazide followed by 1.5% ferrocyanide and 1% osmium for 30 minutes. hAFCs were then counterstained with 0.5% uranyl acetate overnight at 4°C, dehydrated in graded series of ethanol, embedded in Epoxy medium (Epon 812 Fluka, Sigma Aldrich) and polymerized at 60°C for 72 hours. From each sample, ultrathin (55–60 nm thick) sections were cut with a Leica EM UC6 ultramicrotome and examined with an EFTEM equipped with a YAG scintillator slow scan CCD camera (Zeiss).

### FACS

Both control and double-labeled hAFCs were analyzed by cytofluorymetric analysis. Cells were harvested with trypsin, and incubated for 20 minutes at RT with fluorescein-conjugated (FITC)-anti CD105, phycoerythrin-conjugated (PE)-anti CD90, PE-anti CD73 and PE-anti CD146 (all from Becton Dickinson, Franklin Lakes, NJ, USA). After 10 minutes of lysis (FACS Lysing Solution, Becton Dickinson), the samples were centrifuged. The pellets, suspended in 400 µl of PBS, were acquired by a flow cytometer (FACSCanto II Becton Dickinson) and analyzed using Diva software. FITC Annexin V in conjunction with propidium iodide (PI, both from Sigma Aldrich) were used following the manufacturer's instructions. For cell cycle analysis, hAFCs were fixed in 1 ml of 70% ethanol for at least 2 hours, treated with 0.5 mg/ml of RNaseA (Sigma Aldrich) in PBS with 0.1% saponin for 1 hour at 37°C, stained with 20 µg/ml of PI, and analyzed for DNA content. For each sample, at least 5×10^4^ events were collected.

### Viability and proliferation

hAFCs were double-labeled with the SPIOn and Hoechst 33258, as previously described, and both viability and proliferation were assessed at different time points (T0, T3, T7 and T10; T are expressed as days *in vitro*).

Viability of cells was evaluated by a 3-(4,5-dimethylthiazol-2-yl)-5-(3-carboxymethoxyphenyl)-2-(4-sulfophenyl)-2H-tetrazolium (MTS) assay (CellTilter 96® AQueous Assay, Promega, Madison, WI, USA). Labeled cells were plated in 96-well plates at a density of 50,000 cells/well and maintained at 37°C for 1 hour. MTS solution (20 µl/well) was added to cells and incubated for 1 hour at 37°C. The supernatant was then collected, centrifuged at 2,000×g for 5 minutes and read at 490 nm with a microplate reader (Elx800, Bio-Tek Instruments Inc., Winooski, VT, USA).

Maintenance of cell proliferation was tested by plating 750,000 cells/well in quadruplicate in 12-well plates. At each defined time point (T0, T3, T7 and T10) hAFCs were counted and re-plated at the initial concentration to verify any possible difference in proliferative rates between controls and double-labeled hAFCs. For counting, cells were collected, centrifuged at 1,000×g for 10 minutes and suspended in 500 µl of fresh medium. Thereafter, vital cells were counted using the Trypan blue (Sigma Aldrich) exclusion method.

### Animals

Wobbler mice and healthy littermates were bred at Charles River (Calco, Lecco, Italy). Since heterozygous mice do not show any phenotypic difference compared to homozygous healthy littermates, heterozygous founders were determined by genotyping [19].

Procedures involving animals and their care were conducted in conformity with the institutional guidelines that are in compliance with international laws and policies (EEC Council Directive 86/609, OJ L 358, 1 Dec.12, 1987; NIH Guide for the Care and use of Laboratory Animals, U.S. National Research Council, 1996). This specific protocol for the use of laboratory animals was approved by the Italian Ministry of Health and by an internal ethical committee (as stated on the Ethical Committee Approval document (ID.#A5023-01) by the NIH-Office for Protection from Research Risks).

Four weeks old wobbler mice and age-matched healthy littermates, were enrolled for this study. Wobbler mice were easily identified by phenotype features, such as the drastic reduction of growth (in length and body weight) and the appearance of sustained tremors [9].

Wobbler and healthy mice received, under anesthesia, 100,000 double-labeled hAFCs resuspended in 5 µl sterile PBS into the lateral cerebral ventricles (2.5 µl/ventricle, intracerebroventicular administration, ICV) as previously described [6]. All animals daily received cyclosporin A (Sigma-Aldrich) (0.1 mg/ml in drinking water) to avoid rejection of human cells.

To verify if the administration of hACFs alone may modify the hyper-intense signal and lead to important alteration in brain ventricles (e.g. hydrocephalus), four weeks old wobbler mice and healthy littermates also received, under anesthesia, the same number/volume of iron-free hACFs or PBS alone. MRI analyses were carried out 1, 7 and 28 days after graft.

Moreover, to evaluate the clearance of free SPIOn in the ventricular system, four weeks old wobbler mice and healthy littermates received, under anesthesia, 35 µg/ml of SPIOn diluted in 5 µl of sterile PBS by ICV injection. MRI analyses were carried out before SPIOn administration, 1, 24 and 48 hours after ICV injection, as described in *Magnetic Resonance Imaging* section.

The whole experimental schedule is reported in Table 1.

**Table 1:** Experimental groups for *in vivo* analyses.

Group	Days						
	−1	0	1	2	14	28	56
**1** (H; n = 4)	M	L hAFCs	M/S				
**2** (W; n = 4)	M	L hAFCs	M/S				
**3** (H; n = 4)	M	L hAFCs	M		M/S		
**4** (W; n = 4)	M	L hAFCs	M		M/S		
**5** (H; n = 6)	M	L hAFCs	M			M/S	
**6** (W; n = 6)	M	L hAFCs	M			M/S	
**7** (H; n = 4)	M	L hAFCs	M		M		M/S
**8** (W; n = 4)	M	L hAFCs	M		M		M/S
**9** (H; n = 2)	M	U hAFCs	M				M/S
**10** (W; n = 2)	M	U hAFCs	M				M/S
**11** (H; n = 2)	M	PBS	M				M/S
**12** (W; n = 2)	M	PBS	M				M/S
**13** (W; n = 2)	M	Free SPIOn	M	M/S			
**14** (H; n = 2)	M	PBS	M	M/S			

Days are referred as days after transplantation (corresponding to Day 0).

H: healthy mice; W: wobbler mice, M: MRI; L hAFCs: SPIOn/Hoechst 33258 labeled cells; U hAFCs: unlabeled cells; S: sacrifice.

### Magnetic Resonance Imaging (MRI)

MRI experiments were performed on a 7 T/30 cm horizontal bore magnet (Bruker-Biospin, Ettlingen, Germany) equipped with a 12-cm gradient set capable of supplying up to 400 mT/m. T2w images were obtained using a spin-echo MRI sequence (TR/TE = 4000/36 ms, 78 µm^2^ in-plane resolution, FOV 2×2 cm^3^, matrix size = 256×256, slice thickness = 1 mm).

During MRI, the animals were anesthetized by breathing a mixture of 1% isoflurane, 30% O_2_, 70% NO_2_. The animal temperature was kept at 36 +/−1°C using a warmed cradle.

Regions of interest (ROIs) were manually defined, using a mouse brain atlas as a reference [20], with ImageJ software (http://rsbweb.nih.gov/ij/). All the hypo-intense zones were selected and compared to the total brain parenchyma volume. The ratio between the two areas at different time points in healthy and wobbler mice was then compared.

### Histology

To ensure optimal quality for cryostat sections, animals were transcardially perfused as previously described [21].

Free-floating sections (30 µm thick) were incubated with a primary antibody directed against carboxydextran (1∶200, StemCell Technologies) followed by a goat-anti mouse secondary antibody (1∶1,000, Alexa-488, Molecular Probes, Invitrogen) in PBS with NGS. Similarly, a primary monoclonal antibody directed against Human Leucocyte Antigens (HLA)-Class I(1∶500, Immunological Science, Rome, Italy), was utilized to reveal the presence of human cells in the brain ventricles of both healthy and wobbler mice. A secondary anti mouse antibody (1∶1,000, Alexa-546, Molecular Probes, Invitrogen) was utilized to reveal the signal.

Sections were analyzed with a BX81 microscope equipped with a F-view II CCD camera (Olympus). Co-localization between the signals for Hoechst 33258 and Alexa-546 was analyzed by an unmixing fluorescence module to exclude the possible crosstalk between channels.

### Statistics

All values are expressed as mean ± SD. Comparison between groups was made by ANOVA or the Welch's t-test, as specified in the respective figure/table legends, using a dedicated statistical software (GraphPadPrism Inc. La Jolla, CA, USA). The minimum level of statistical significance was set at p<0.05.

## Results

### *In vitro* characterization

**Figure 1:**
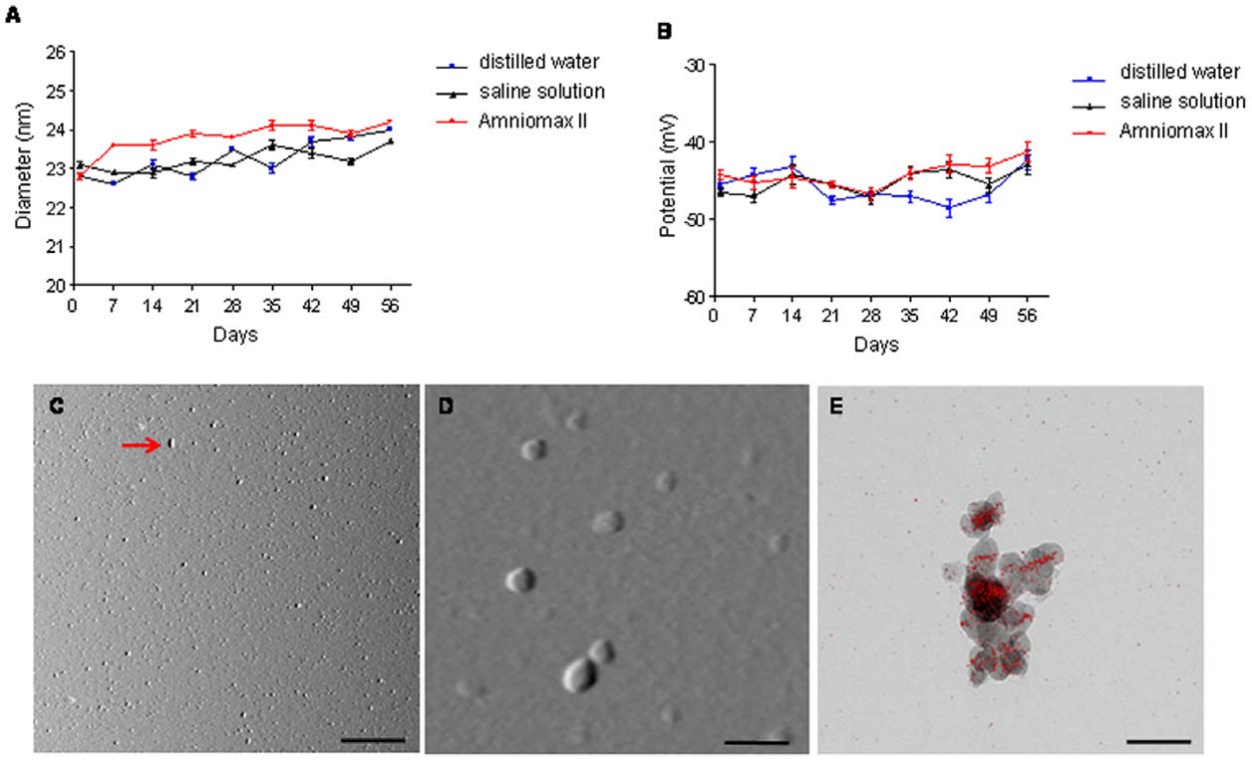
*In vitro* measurements of SPIOn. (**A**) Size distribution and (**B**) zeta-potential measured in distilled water, saline solution and Amniomax II by DLLS. (**C–D**) Representative images of SPIOn spotted on the mica and visualized by AFM. (**C**) SPIOn are mainly monodispersed although a small number of chain-like clusters (red arrow) were found. (**D**) AFM picture shows the spheroid shape of SPIOn. (**E**) EFTEM images of SPIOn. Single particles were detectable, despite aggregation caused by magnetic forces, confirming data obtained by AFM. Red spots indicate the ESI analysis for iron. Scale bars: C = 1 µm; D–E = 40 µm.

The weekly DLLS measurements (Figure 1, *A* and *B*) showed that neither the prolonged incubation (56 days) nor the different type of medium modified the size, the polidispersity index and the zeta potential of SPIOn. The visualization by AFM confirmed that SPIOn are monodispersed and homogeneous for size/shape (Figure 1, *C* and *D*). The presence of iron domains in SPIOn is shown in Figure 1, *E* (red spots enclosed in gray spheres). It is of interest to notice that red spots are not homogeneously distributed in SPIOn but seem to align by forming a magnetic field. All these experiments were important to verify the stability of SPIOn in biological samples.

### Tracer internalization

**Figure 2:**
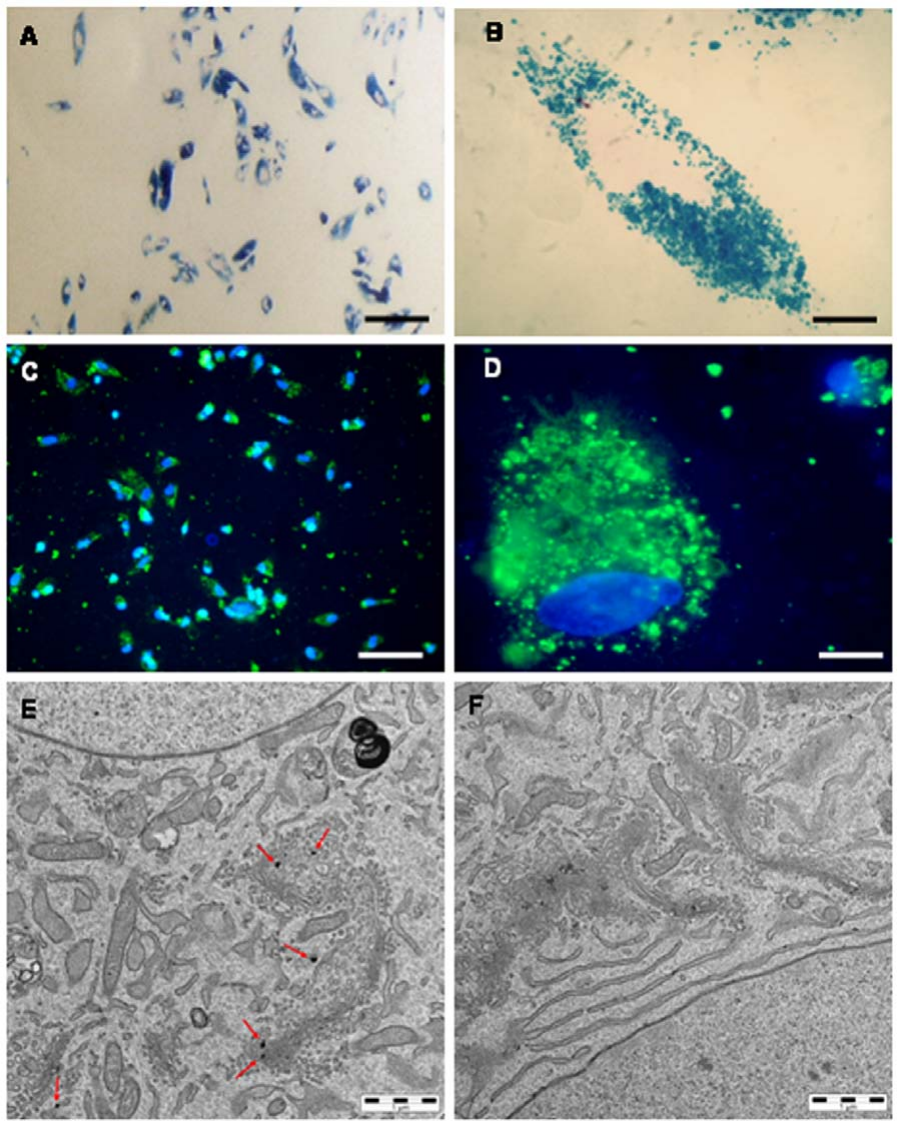
Detection of SPIOn internalization in hAFCs. (**A**) Representative picture of Prussian blue staining. Internalization process of SPIOn in hAFCs involved the whole cell population regardless the different morphologies. (**B**) Higher magnification figure of Prussian blue staining. (**C**) Representative figure of Hoechst 33258 (blue) and anti-carboxydextran staining (green). (**D**) Higher magnification picture of carboxydextran and Hoechst 33258 internalization. (**E**) Aggregates of SPIOn not enclosed by a membrane in the cytoplasm of hAFCs (red arrows). (**F**) TEM analysis in iron-free hAFCs (F). Scale bars: A–C = 100 µm; B–D = 20 µm. E = 1 µm.

Prussian blue staining revealed a high efficiency of SPIOn internalization into hAFCs 72 hours after incubation (Figure 2, *A*). High magnification picture showed a selective staining in the cytoplasm (Figure 2, *B*). Such feature was confirmed by the co-localization experiments between carboxydextran immunostaining (green) and the nuclear staining by Hoechst 33258 (blue) (Figure 2, *C* and *D*). Unlabeled hAFCs did not show any positive staining for either tracer (not shown).

TEM showed small clusters of SPIOn not surrounded by a membrane (Figure 2, *E*, red arrows) in the intracellular space close to the perinuclear area, but not inside the nuclei. The presence of SPIOn did not modify the morphology of either organelles (endoplasmic reticulum, Golgi apparatus, mitochondria, endosomes) and cellular membranes of hAFCs, as confirmed by the comparison with the ultra structure of iron-free cells (Figure 2, *F*).

### Viability and proliferation

**Figure 3:**
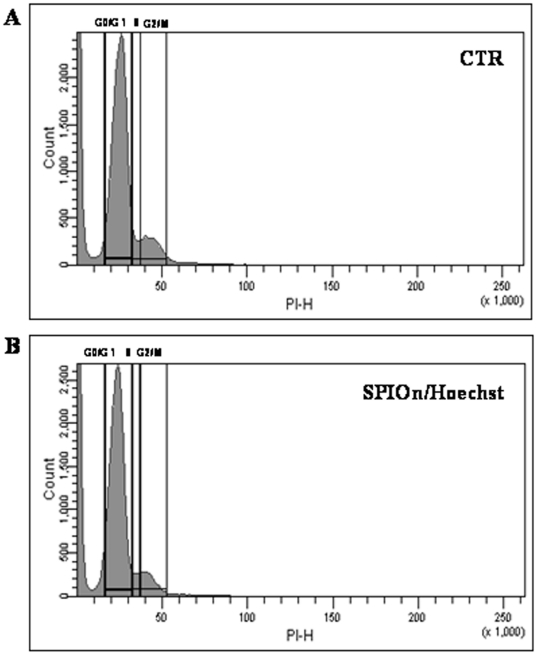
FACS analysis of hAFCs. Evaluation of cell cycle revealed that there were no differences between unlabeled (**A**) and SPIOn/Hoechst 33258 positive hAFCs (**B**). In all, 50,000 events per histogram were analyzed (horizontal axis: linear fluorescence intensity; vertical axis: relative cell number). Additional data are reported in Table 2.

**Figure 4:**
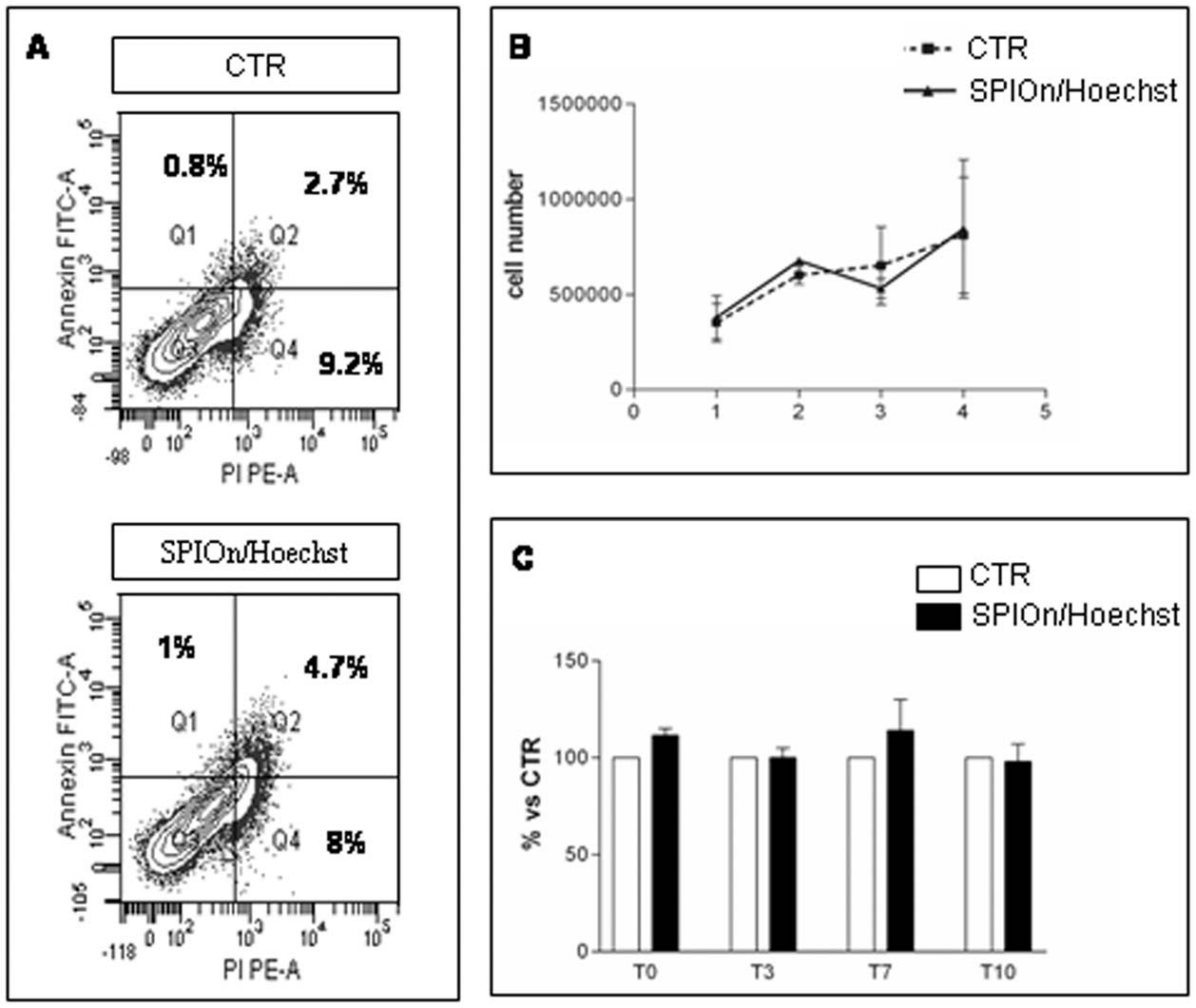
Evaluation of vital parameters in labeled hAFCs. (**A**) Representative figure of apoptosis measurement by Annexin V/PI staining and FACS analysis. The lower left fractions of each panel indicate viable cells (Annexin negative-PI negative, Q3). Early (Annexin positive-PI negative, Q1) and end stage (Annexin positive- PI positive, Q2) apoptotic cells are in the upper left and upper right fractions respectively. Dead cells (Annexin negative-PI positive, Q4) are in the lower right fractions. No significant differences were found between the two experimental groups (see also Table 2). (**B**) Proliferation of hAFCs. SPIOn and Hoechst 33258 internalization did not influence the proliferative capability of cells. T is expressed as days *in vitro*. Data are mean ± SD from 2 replicates of 3 different experiments. Statistical analysis: Two-Way ANOVA and Bonferroni post test for multiple comparisons. (**C**) Metabolic activity assessment. MTS assay showed no differences between control and SPIOn/Hoechst 33258 labeled hAFCs, at either T0 or 10 days after labeling. Data are expressed as mean ± SD from 2 replicates of 3 different experiments. Statistical analysis: Two-Way ANOVA and Bonferroni post test for multiple comparisons.

SPIOn/Hoechst 33258 labeling in hAFCs did not lead to significant changes either in the cell cycle or in the percentage of cell surface markers. Representative results are shown in Figure 3 and summarized in Table 2. No statistically significant difference between groups was observed. Moreover, SPIOn internalization did not increase the percentage of Annexin V/PI positive cells (Figure 4, *A*). The simultaneous internalization of SPIOn and nuclear labeling did not alter physiological parameters of hAFCs: at different time (T0, T3, T7 and T10) there was no significant difference in proliferative rate between controls and double-labeled hAFCs (Figure 4, *B*). Furthermore, MTS assay revealed that metabolism of double-labeled hAFCs was comparable to control, indicating that SPIOn is not toxic for cells (Figure 4, *C*).

**Table 2:** Cytofluorimetric results comparing marker expression, apoptotic/dead cells and number of hAFCs in the different cell cycle phases between controls (unlabeled) and SPIOn/Hoechst 33258 labeled cells.

	Unlabeled hAFCs	Labeled hAFCs
**PHENOTYPE**		
**CD105+**	14.03±10.7	13.43±9.9
**CD90+**	50.23±15.6	47.2±12.6
**CD73+**	86±4.9	79.77±11.5
**CD 146**	61.16±30.3	45.87±29.5
**APOPTOSIS AND DEATH**		
	4.2±3.3	5±3.1
**CELL CYCLE**		
**G2**	55±5	52±0.5
**S**	1.9±0.9	1.7±0.9
**G0G1**	7±1.6	5.5±0.05

Data are expressed as the percentage of three independent experiments ± SD. Statistical analysis: Welch's t-test.

### *In vivo* visualization of hAFCs and free SPIOn in cerebral ventricles of transplanted mice

**Figure 5:**
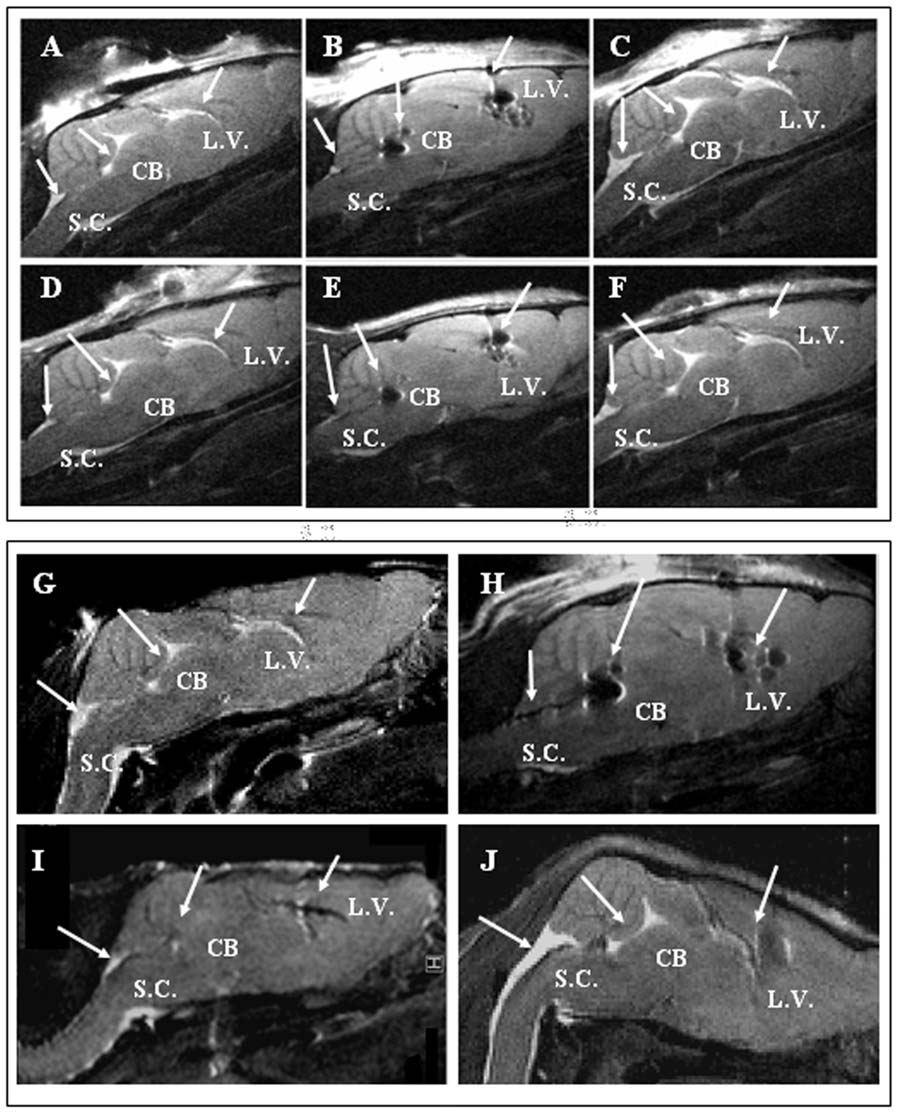
Representative longitudinal MRI of wobbler mouse brains 1 day after ICV administration of PBS (A), hAFCs labeled with SPIOn (B) and iron-free hAFCs (C). Representative longitudinal MRI of the same mouse brain 28 days after ICV administration of PBS (**D**), hAFCs labeled with SPIOn (**E**) and iron-free hAFCs (**F**). Arrows indicate the different compartments of brain ventricles: L.V.: lateral ventricle; CB: cerebellum; S.C.: spinal cord. Representative longitudinal MRI of wobbler mouse brains before (**G**), 1 (**H**), 24 (**I**) and 48 hours (**J**) after SPIOn administration. For each panel, the sagittal slice is representative of the region comprising lateral ventricles (L.V.), cerebellum (CB) and fourth ventricle at the spinal cord level (S.C.), as indicated by arrows.

T2 weighed MRI coronal brain slices showed a diffused light gray shade representing the brain parenchyma whereas a brighter hyper-intense signal was associated with brain ventricles (Figure 5, *A*). A hyper-intense signal was observed in the ventricular system of wobbler mice receiving PBS alone (Figure 5, *A*, see arrows) one day after ICV, while a hypo-intense signal was observed in animals receiving hACFs loaded with SPIOn (Figure 5, *B*, see arrows). Conversely, the hyper-intensity was preserved after unlabeled hACF graft (Figure 5, *C*, see arrows).

The MRI signal still remained comparable between 1 and 28 days after administration of PBS (Figure 5, *D*), SPIOn-labeled hACFs (Figure 5, *E*) or iron-free hAFCs (Figure 5, *F*),

Interestingly, the comparison between animals treated with PBS (Figure 5, *A* and *D*), labeled (Figure 5, *B* and *E*) and iron-free hAFCs (Figure 5, *C* and *F*) did not show any differences in the morphology of the ventricular compartments for at least 28 days after ICV transplantation, irrespectively of the pathology.

Before SPIOn injection (untreated animals) a strong hyper-intense signal was clearly visible in the ventricular systems (Figure 5, *G*, see arrows) similarly to animals treated with PBS alone (Figure 5, *A*). Traceability, distribution and half-life of SPIOn in the lateral ventricles of a four weeks old wobbler mouse at different time points is shown in Figure 5 *H*–*J*.As expected, 1 hour after ICV administration of SPIOn, the hypo-intense signal rapid spread in the whole ventricular system, as revealed by dark regions in different compartments (Figure 5, *H*, see arrows). In contrast to the persistent signal retrieved after SPIOn labeled-hAFC graft (see Figure 5, *E*, see arrows), the hypo-intense signal rapidly decayed in animals receiving SPIOn alone (Figure 5, *I*, see arrows). Twenty-four hours after ICV, hypo-intense areas associated with lateral ventricles, cerebellum and cervical spinal cord were indeed drastically reduced if compared to Figure 5, *H*. Forty-eight hours after SPIOn administration, MRI images showed a very mild area of hypo-intense signal in correspondence to the site of administration (Figure 5, *J*, see arrow in L.V.) whereas in the other regions the signal was similar to that observed before their injection (Figure 5, *G*). Our study did not reveal any different SPIOn behavior between healthy and wobbler mice.

### *In vivo/ex vivo* hAFC tracking

**Figure 6:**
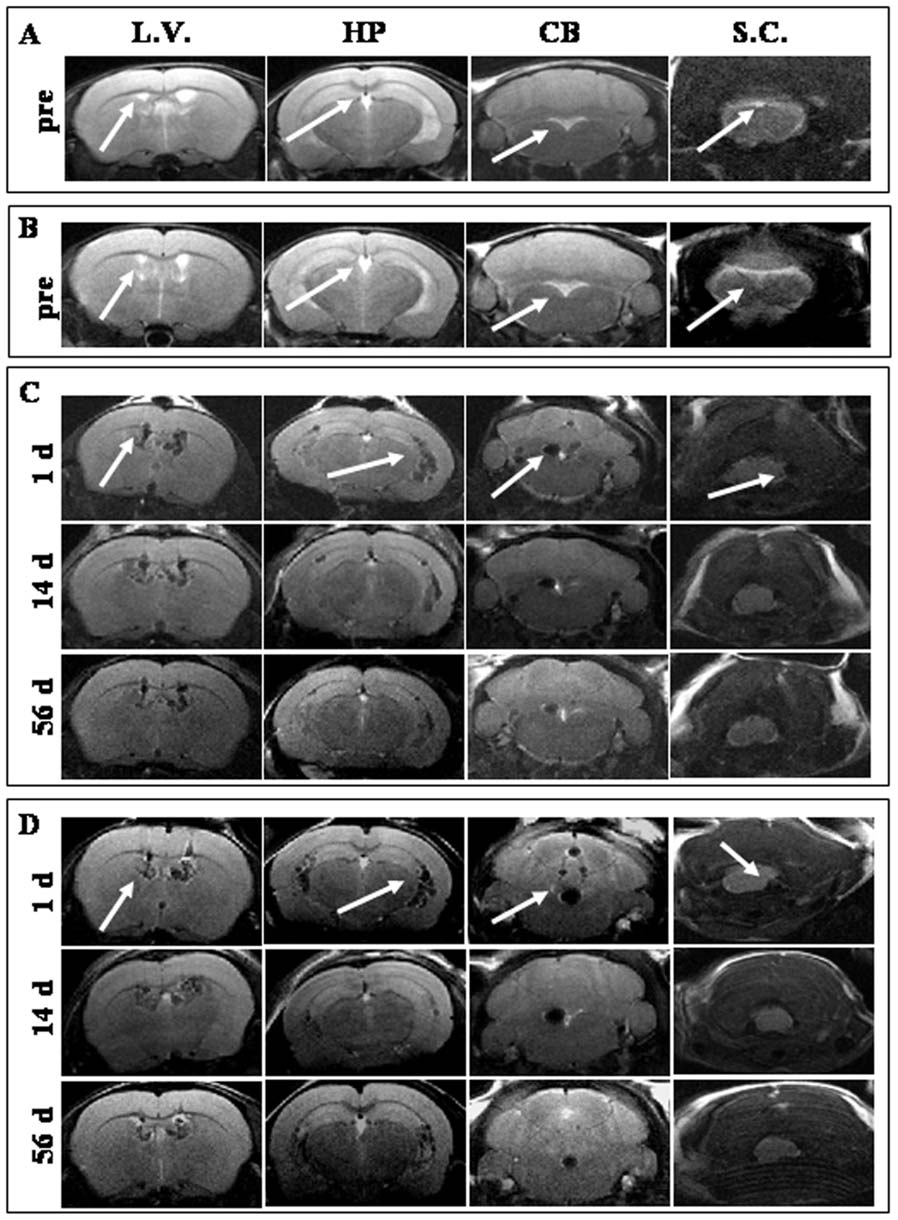
MRI of labeled hAFCs. Representative pictures of MRI of healthy (A) and wobbler (B) mice one day before hAFC transplantation. Axial MRI analysis of the same healthy (**C**) and wobbler mice (**D**) at 1, 14 and 56 days after graft. For each panel, the coronal slices are indicative of different regions of the ventricular system including the site close to cell administration (L.V.), the region corresponding to the ventral hippocampus (HP), the region between the brainstem and the cerebellum (CB) and the cervical spinal cord region (S.C.).

MRI, performed one day before hAFC transplantation, showed a marked hypo-intense signal both in healthy (Figure 6, *A*, see arrows) and wobbler mice brains (Figure 6, *B*, see arrows).

**Figure 7:**
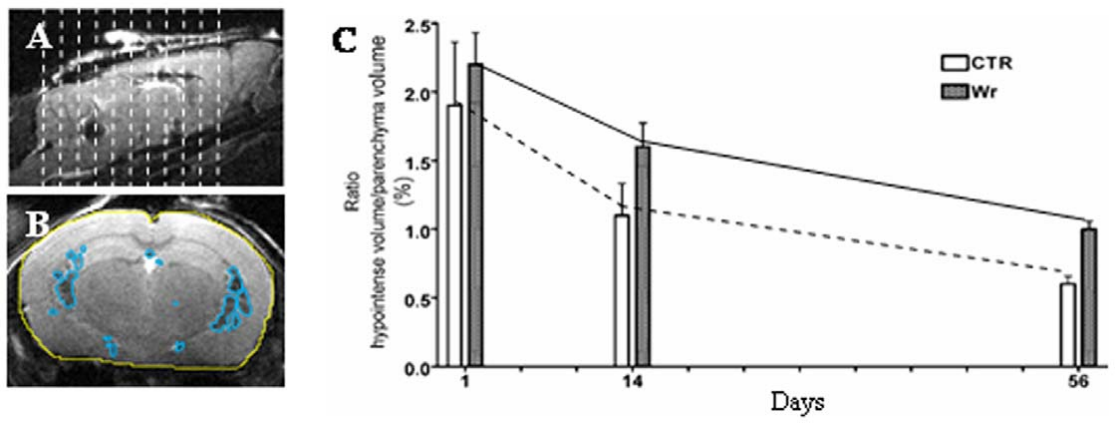
Relative quantification of MRI signal. (**A**) Acquisition scheme of serial slices selected for the quantification of volumes. (**B**) Representative slice showing the ROI manually defined for the volume quantification. Light blue areas show the hypo-intense signal, while yellow area show the brain parenchyma. (**C**) Quantification of the percentage of hypo-intense volume in the brain. No significant difference was found between wobbler mice and healthy controls 1, 14 and 56 days after hAFC transplantation. Data are expressed as mean ± SD (n = 4) for each group. Statistical analysis: Two-Way ANOVA and Bonferroni post test for multiple comparisons.

One day after SPIOn loaded-hAFC administration, the signal in the brain ventricles became darker and hypo-intense in both healthy (Figure 6, *C*, upper panels, see arrows) and wobbler mice (Figure 6, *D*, upper panels, see arrows). This effect rapidly spread along the whole ventricular system in both experimental groups and reached the fourth ventricle around the cervical spinal cord. On the contrary, MRI from animals receiving iron-free hAFCs did not reveal any modification on the hyper-intensity of signal associated with the cerebrospinal fluid (CSF). Fourteen days after hAFC graft, in both healthy (Figure 5, *C*, middle panels) and wobbler mice (Figure 5, *D*, middle panels), the pattern of hypo-intense signal was not markedly reduced, did not modify its distribution in ventricles and did not seem to spread to the brain parenchyma. The hypo-intense signal in the brain ventricles of both experimental groups decayed 56 days after transplantation (Figure 6, *C* and *D*, lower panels) while remaining detectable in the whole ventricular system. Serial MRI slices corresponding to the ventricular system (Figure 7, *A*) were used to perform a longitudinal measurement of hAFC distribution in both wobbler and healthy mice (n = 4 for each group) by calculating the ratio between the volume of hypo-intense signal (Figure 7, *B*, light blue regions) and the total brain parenchyma volume (Figure 7, *B*, yellow area). A progressive decrease of hypo-intense volume occurred in both experimental groups, but no relevant difference was found by comparing the signal decay over time (Figure 7, *C*).

**Figure 8:**
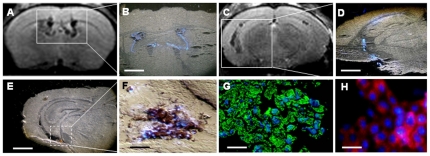
*Ex vivo* analyses. (**A**) MRI pictures showing axial slices corresponding to the lateral ventricle close to the site of administration. (**B**) Histological section observed by UV shows the presence of Hoechst 33258 positive nuclei in the ventricular area. (**C**) MRI axial slice close to the ventral hippocampus. (**D**) Fluorescent microscopy of histological section revealed a co-localization between Hoechst 33258 and MRI signal. (**E**) Iron accumulation in the brain section adjacent to that shown in figure D. (**F**) High magnification picture showing the merge between iron accumulation and Hoechst 33258 positive nuclei. (**G**) Merge between the carboxydextran immunoreactivity (green) and Hoechst 33258 positive nuclei (blue). (**H**) Merge between the HLA-I (red) and Hoechst 33258 positive nuclei (blue). Scale bars: B–D–E = 600 µm; F–G = 45 µm; H = 30 µm.

This evidence was also a confirmed by *ex vivo* studies (Figure 8, *A–D*). The MRI coronal slice from a wobbler mouse, 28 days after cell graft, showed a hypo-intense signal in the lateral and third ventricles (Figure 8, *A*). The corresponding histological section (the mouse was sacrificed few minutes after MRI) showed the presence of Hoechst 33258 positive nuclei exclusively confined to the ventricular system and overlapping with the MRI hypo-intense signal (Figure 8, *B*). A similar pattern of hypo-intense MRI signal (Figure 8, *C*) and Hoechst 33258 positive nuclei (Figure 8, *D*) was found in the lateral ventricles close to the ventral hippocampus. High magnification imaging of an adjacent brain section (Figure 8, *E*) showed a cellular co-localization between the brown staining associated with iron accumulation (cytoplasmic) and the Hoechst 33258 staining (nuclear) (Figure 8, *F*). The merge of the carboxydextran immunoreactivity (green staining) and the Hoechst 33258 in the following brain section, confirmed that iron accumulation was actually associated with SPIOn (Figure 8, *G*). Moreover, the co-localization experiment between Hoechst 33258 and HLA-I (Fig. 8 *H*), confirmed that almost all the Hoechst 33258-positive cells in the ventricular system also expressed the human endogenous marker.All these data reveal the very high correlation between the nuclear tracer Hoechst 33258 and: 1) the hypo-intense signal from MRI; 2) the dark brown deposition (typical of iron oxides); 3) the carboxidextran (coating agent of SPIOn); 4) the human endogenous marker HLA-I. Altogether, our experimental evidence strongly confirmed the reliability of Hoechst 33258 labeling, thus enabling us to directly track hAFCs by the evaluation of positive nuclei in the brain and spinal cord of healthy and wobbler mice at different times after transplantation (see *Text S1* and *Figure S1*).

All recruited animals survived for the whole duration of the study, suggesting that the ICV transplantation of SPIOn/Hoechst 33258 labeled hAFCs did not lead to acute toxicity in either wobbler or healthy mice. In addition, the lack of apparent alteration to ventricular morphology (see Figure 5, *A*–*F*), and the very weak expression of markers for leukocyte infiltration (see *Text S1* and *Figure S2*), further supports the hypothesis of a safe and reliable approach by these two fluo-paramagnetic tracers in hAFC ICV transplantation.

## Discussion

In this study, we described for the first time the paramagnetic labeling of hAFCs and their subsequent long-term tracking in a chronic neuro-degenerative/inflammatory environment, such as wobbler mice brains. We demonstrated that hAFC survival was not altered by the presence of double tracers, permitting substantial correlations between *in vivo* and *ex vivo* data at different times. Finally, the overall result emerging from our experiments suggests that this is a reliable strategy able to combine a high and satisfactory level of biocompatibility with an efficient and long lasting cell labeling.

Initially, we focused our attention on the chemico-physical properties of SPIOn when incubated in different media, to exclude possible interactions with biological proteins and to reproduce the conditions that SPIOn encountered during the process of cell internalization. DLLS, AFM and ESI analyses did not suggest any important alteration of SPIOn stability for at least 56 days. This result is in accordance with previous studies performed by DLLS [22].

Once we had ascertained SPIOn stability over time, we verified the dynamics of their uptake and the consequent sub-cellular distribution.

On the basis of data in the literature [23], we decided to utilize a 35 µg/ml concentration of SPIOn which allows 100% labeling efficiency, even in rapidly proliferating adhesive cells, such as hAFCs, avoiding the toxic effects related to higher SPIOn content. Prussian blue and carboxydextran staining confirmed the ability of SPIOn to be internalized into cells and to selectively spread in the cytoplasm [13]. The visualization by TEM of cytoplasmic small iron aggregates not surrounded by membranes, suggested that SPIOn internalization into hAFCs is based on a non-endocytic mechanism or an endosomolytic uptake [24] and did not alter sub-cellular morphology. Moreover, the lack of significant differences in viability and functionality between double-labeled and control cells further confirmed that neither cell phenotype, nor replication were influenced by the presence of SPIOn [25]. In view of potential clinical applications, it is also noteworthy that iron labeling does not alter cell division, providing some reassurance about their possible tumorigenic potential [26].

In addition, simultaneous internalization of SPIOn and Hoechst 33258 did not affect proliferation and metabolic activity in hAFCs. In agreement with published data [23], [27], these parameters appeared similar to control cells at the different time-points considered in the study. The slight increase in metabolic activity at T0 and T7 observed in cells loaded with SPIOn could be related to the higher iron concentration available for biological processes inside cells, as suggested by Arbab and colleagues [27]. The lack of SPIOn toxicity upon internalization was further confirmed by Annexin V/PI staining which excluded different apoptotic/death rates in cells, concordantly with previous publications [26].

We did not observe adverse side effects after double-labeling with regard to both animal and cell distribution, further underlining the safety of the procedure. Our main findings were that: 1) the rate of mortality, monitored over the 56 days after transplantation, was indeed very low and did not differ from the animals receiving PBS/unlabeled hAFCs, and 2) macroscopic features of inflammatory response and rough modification of the ventricular system were not observed in either wobbler or healthy mice throughout the duration of our study. These results seem to confirm the reliability of labeled hAFCs for future investigation of therapeutic efficacy in models of neurological disorders.

In the last decade, a large number of studies have been published regarding the *in vivo* tracking of different SC types loaded with SPIOn, administered by several routes, in different pathological conditions [26]. The MRI analysis we performed in healthy and wobbler mice receiving hAFCs labeled with multi-modal exogenous tracers, revealed that the signal rapidly spread up in/to the spinal cord, remained confined to the ventricles, and decayed over time, but was still detectable up to the endpoint of our study.

As previously documented [28], a combined *in vivo* and *ex vivo* study was carried out in mice at different times after hAFC graft to establish whether the hypo-intense signal observed in brain ventricles was actually associated with the fluorescent tracer. The visualization of a large number of Hoechst 33258 positive nuclei in relation to the MRI signal confirmed the presence of transplanted cells. Although some leakage of the nuclear dye from grafted cells to host tissue remains possible [29], the co-localization of Hoechst 33258 with either the carboxydextran or anti HLA supports our findings.

In the literature, the issue of SPIOn leakage has already been investigated: Flexman et al., [30] demonstrated that the MRI signal was still detectable one week after intraparenchymal injection of free tracer. Conversely, we demonstrated that within the first 24 hours, the pattern of hypo-intense signal for free SPIOn (35 µg/ml), injected in mice by ICV, was comparable to the distribution of transplanted iron-positive cells. However, in the following 48 hours, the signal rapidly faded and completely disappeared. This difference could be due to the rapid flow of CSF across the ventricular system that allows a fast clearance of free tracer. Altogether the data presented here support the hypothesis of SPIOn persistence inside viable cells for the whole longitudinal study in our experimental conditions

It has already been demonstrated that the relative quantification of SPIOn-labeled cells by MRI is feasible and useful for studying cell migration through specific tissues [28]. However the large majority of measures were carried out using labeled cells grafted in parenchymal tissue after acute damage [31], [32] while our comparison was conducted for the first time in a chronic model of disease with labeled hAFCs directly injected in the ventricular system. Concordantly, our quantification did not reveal any important difference in the anatomical/temporal cell pattern, in either healthy or wobbler mice.

It is important to underline that the therapeutic effect in different chronic (neuro)degenerative disorders is now predominately associated with SC permanence and the release of several factors by transplanted cells, even far from the site of injury [3], [33]. As a matter of fact we have already demonstrated that ICV injection of SCs in two different ALS animal models (SOD1G93A and wobbler mice) resulted in significant therapeutic improvements although no cell localization in the pathological tissue was observed [6]. Moreover, intrathecal and intravenous SC administration have already been tested in a preliminary clinical trial aimed at demonstrating the safety and immunological effects of mesenchymal SC transplantation in patients with multiple sclerosis and ALS [34]. Direct (multiple) injection in the brain ventricles is less invasive than intraspinal administration and is capable of extensively delivering cells *via* the CSF [35] with therapeutic improvements devoid of side-effects [36]. On the other hand, no evident clinical benefits have been detected so far in ALS patients after intraspinal delivery of autologous mesenchymal SCs [37]. The data presented here demonstrate that hAFCs rapidly diffuse throughout the forth ventricle at the level of the spinal cord, however, they did not migrate into the parenchyma. Survival and long-term spreading of hAFCs appeared particularly intriguing since cell diffusion from the brain towards damaged areas become exploitable to avoid invasive intraspinal implantation of SCs in patients.

Altogether our data describe for the first time the potential of ICV transplantation and long-term SC imaging in a mouse model of ALS, characterized by an ongoing degenerative environment. A final novel point which emerged from this work is the concomitant employment of a nuclear-fluorescent marker with a well-characterized super paramagnetic cytoplasmic tracer in SCs obtained from fetal tissues.

Additional preclinical trials, aimed at coupling the tracking of SPIOn-labeled hAFCs to their possible therapeutic effect are however, required to elucidate their potential in clinical therapy for motor neuron disorders.

## Supporting Information

Figure S1 **Tracking of Hoechst 33258 positive hAFCs in transplanted brain of wobbler mouse.** (**A–F**) Distribution of Hoechst 33258 labeled cells in the lateral ventricles close to the site of hAFC injection (A), the choroid plexus (B), the 3^rd^ ventricle (C), the lateral ventricles close to the ventral hippocampus (D), the brainstem (E) and the spinal cord (F) 1 day after hAFC transplantation. Scale bar: A, B, F = 200 µm; C, D = 150 µm; E = 120 µm. (**G–J**) Localization of Hoechst 33258 positive nuclei 14 days after hAFC graft. Scale bar: G, H, I, K = 150 µm; J = 120 µm. (**L**) High magnification picture shows a cluster of Hoechst 33258 positive nuclei attached to the meningeal layer at the cervical spinal cord level. Scale bar = 40 µm. (**M–Q**) Localization of Hoechst 33258 positive hAFCs 28 days after transplantation. Scale bar: M, P, Q = 200 µm; N, O = 150 µm. (**R–U**) Distribution of Hoechst 33258 positive nuclei in lateral ventricles (R, S), third ventricle (T) and brainstem (U) 56 days post grafting. Scale bar = 150 µm. (**V**) High magnification picture showing a group of Hoechst 33258 positive nuclei in the ventral region of white matter at the cervical spinal cord level. Scale bar = 40 µm. L.V.: lateral ventricle; C.P.: Choroid Plexus; 3V: 3^rd^ ventricle); HP: Hippocampus; CB: cerebellum; AQ: aqueduct; BS: brainstem; S.C.: spinal cord.
(TIF)

Figure S2 **Analysis of leukocyte infiltration.** (**A–C**) Representative pictures showing the immunoreactivity for CD3 (A), CD4 (B) and CD8 (C), in lateral ventricles of wobbler mouse brain 28 days after PBS administration. (**D–F**) Representative pictures showing the immunoreactivity for CD3 (D), CD4 (E) and CD8 (F), in lateral ventricles of wobbler mouse brain 28 days after administration of SPIOn labeled hAFCs. (**G–I**) Representative pictures showing the immunoreactivity for CD3 (G), CD4 (H) and CD8 (I), in spleen sections 28 days after PBS administration in brain lateral ventricles. Scale bar: (A–F) = 40 µm; (G–I) = 30 µm.
(TIF)

Text S1 (DOC)
