# Supplementary figures and images for: Longitudinal Tracking of Human Fetal Cells Labeled with Super Paramagnetic Iron Oxide Nanoparticles in the Brain of Mice with Motor Neuron Disease
Source: PLoS One. 2012 Feb 27;7(2):e32326. doi: 10.1371/journal.pone.0032326 (PMC3288077; doi:10.1371/journal.pone.0032326)

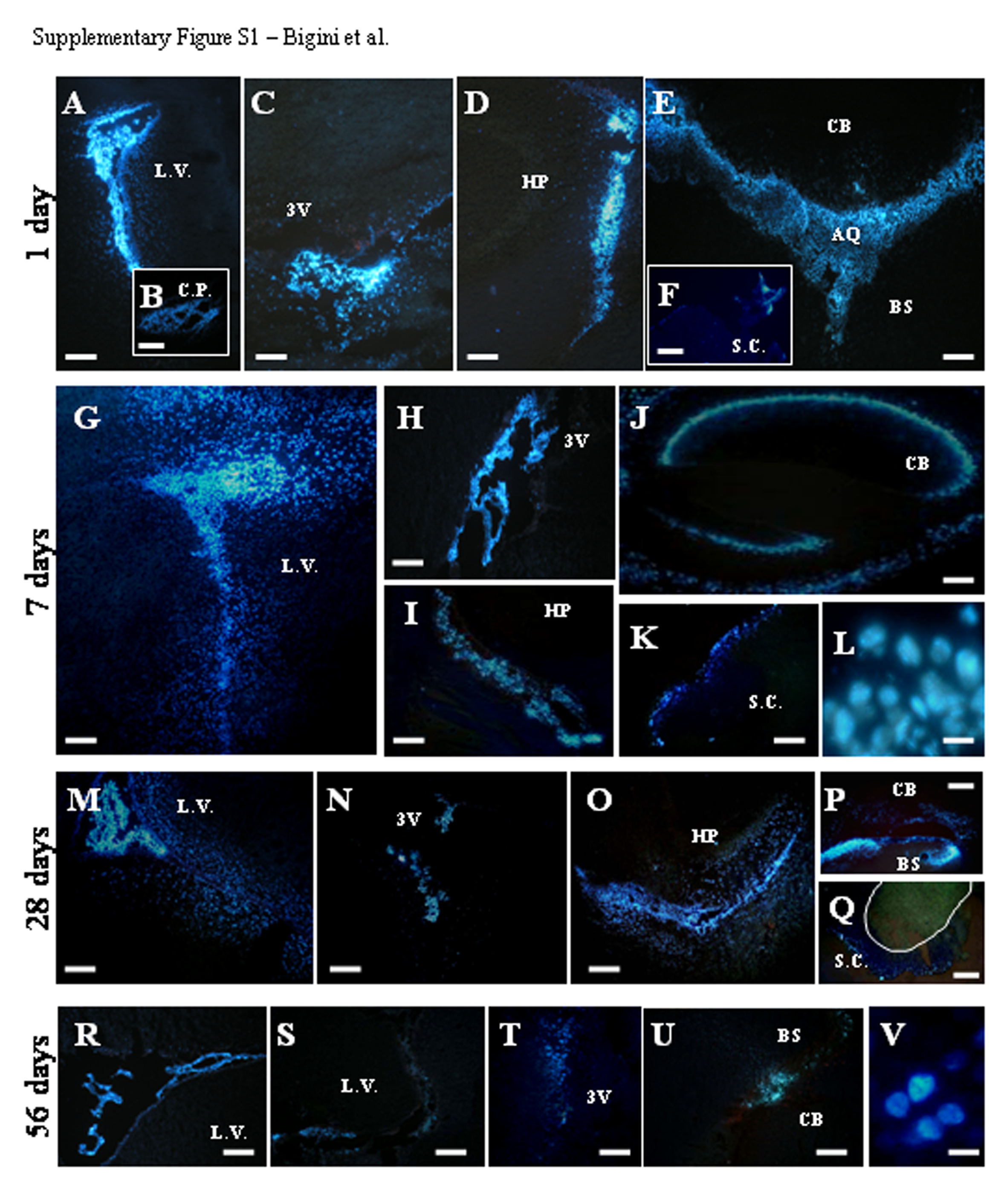

Supplement: Figure S1 — Tracking of Hoechst 33258 positive hAFCs in transplanted brain of wobbler mouse. (A–F) Distribution of Hoechst 33258 labeled cells in the lateral ventricles close to the site of hAFC injection (A), the choroid plexus (B), the 3rd ventricle (C), the lateral ventricles close to the ventral hippocampus (D), the brainstem (E) and the spinal cord (F) 1 day after hAFC transplantation. Scale bar: A, B, F = 200 µm; C, D = 150 µm; E = 120 µm. (G–J) Localization of Hoechst 33258 positive nuclei 14 days after hAFC graft. Scale bar: G, H, I, K = 150 µm; J = 120 µm. (L) High magnification picture shows a cluster of Hoechst 33258 positive nuclei attached to the meningeal layer at the cervical spinal cord level. Scale bar = 40 µm. (M–Q) Localization of Hoechst 33258 positive hAFCs 28 days after transplantation. Scale bar: M, P, Q = 200 µm; N, O = 150 µm. (R–U) Distribution of Hoechst 33258 positive nuclei in lateral ventricles (R, S), third ventricle (T) and brainstem (U) 56 days post grafting. Scale bar = 150 µm. (V) High magnification picture showing a group of Hoechst 33258 positive nuclei in the ventral region of white matter at the cervical spinal cord level. Scale bar = 40 µm. L.V.: lateral ventricle; C.P.: Choroid Plexus; 3V: 3rd ventricle); HP: Hippocampus; CB: cerebellum; AQ: aqueduct; BS: brainstem; S.C.: spinal cord. (TIF) [file pone.0032326.s001.tif]

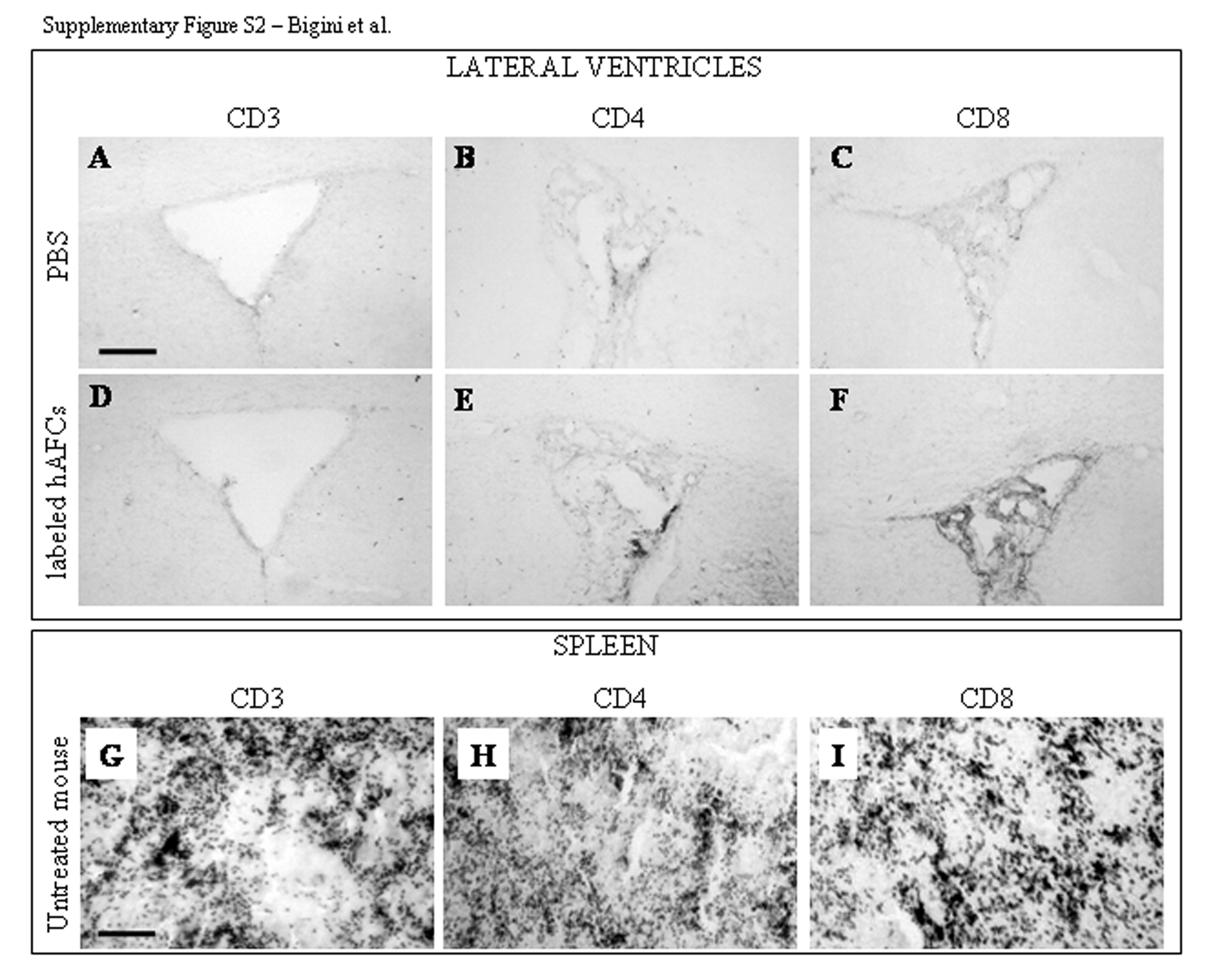

Supplement: Figure S2 — Analysis of leukocyte infiltration. (A–C) Representative pictures showing the immunoreactivity for CD3 (A), CD4 (B) and CD8 (C), in lateral ventricles of wobbler mouse brain 28 days after PBS administration. (D–F) Representative pictures showing the immunoreactivity for CD3 (D), CD4 (E) and CD8 (F), in lateral ventricles of wobbler mouse brain 28 days after administration of SPIOn labeled hAFCs. (G–I) Representative pictures showing the immunoreactivity for CD3 (G), CD4 (H) and CD8 (I), in spleen sections 28 days after PBS administration in brain lateral ventricles. Scale bar: (A–F) = 40 µm; (G–I) = 30 µm. (TIF) [file pone.0032326.s002.tif]
