# Supplementary material for: Longitudinal Tracking of Human Fetal Cells Labeled with Super Paramagnetic Iron Oxide Nanoparticles in the Brain of Mice with Motor Neuron Disease
Source: PLoS One. 2012 Feb 27;7(2):e32326. doi: 10.1371/journal.pone.0032326 (PMC3288077; doi:10.1371/journal.pone.0032326)
Supplement: Text S1 — (DOC) [file pone.0032326.s003.doc]

**Supporting Information S1**

**Materials and methods**

*Hoechst 33528 visualization*

Coronal sections (15 μm thick) were visualized by using different strategies (phase contrast, bright

field, dark field, UV excitation) to determine the histological localization of Hoechst 33258 positive nuclei in different region over time.

*Lymphocytes Immunohistochemistry*

Four weeks old wobbler mice and healthy littermates received, under anesthesia, 5 µl of sterile PBS

(2,5 µl/each ventricle) into the lateral cerebral by ICV injection as described in material and methods section (see *Animals*). A further group of animals was similarly injected with double-labeled hAFCs resuspended in sterile PBS. Twenty-eight days after ICV all animals were sacrificed by intracardial perfusion (see Materials and Methods section). In order to reveal the presence of lymphocytes in brain and spleen sections we performed immunohistochemistry with mouse primary monoclonal antibodies directed against CD3, CD4 and CD8 (1:100, Novocastra Laboratories, Newcastle, UK). A secondary biotinylated antibody directed against mouse (1:200, Vector Laboratories, Burlingame, CA, USA) was used for all sections. Thereafter, sections were incubated for 1 hour in a solution containing reagent A (avidin) and reagent B (biotinylated horseradish peroxidase) (ABC Kit, Vector Laboratories) which allows to visualize the specific antigens.

**Results**

*hAFC tracking by Hoechst 33258 visualization*

The distribution of Hoechst 33258 positive nuclei in the brain ventricles 1 day after hAFC administration confirmed the MRI analysis and further suggests that hAFCs, once transplanted in the anterior body of lateral ventricles (Figure S1, *A*), rapidly diffuse to the other structures of the ventricular system. Hoechst 33258 positive nuclei were found in the choroid plexus of lateral ventricles (Figure S1, *B*), in the third ventricle (Figure S1,*C*), in the lateral ventricles close to the hippocampus (Figure S1, *D*), in the forth ventricle between the cerebellum and the brainstem inside the aqueduct (Figure S1, *E*). In contrast to the intense loss of signal observed at the spinal cord level of both wobbler mice and healthy controls 1 day after hAFC transplantation, the presence of Hoechst 33258 positive in cervical spinal cord section was quite sporadic and only associated with meningeal layer attached to the spinal cord (Figure S1, *F*).

Seven days after hAFC transplantation the amount of Hoechst 33258 positive nuclei close to the side of administration was very high and not only confined to the ventricular layers (Figure S1, *G*). The co-localization experiments with the antibody anti-dextran gave the same results showed in Figure 8, *G*, thus confirming the presence of hAFCs in the brain parenchyma. In both experimental animal models the visualization of the different brain regions did not show relevant difference compared to the animals sacrificed 1 day post transplantation. Many Hoechst 33258 positive cells were found at the third ventricle (Figure S1, *H*), in lateral ventricles close to the hippocampus (Figure S1, *I*), in the fourth ventricle and inside the fissures of cerebellum (Figure S1, *J*). A relevant increase of Hoechst 33258 was instead observed at the cervical spinal cord level, close to the external surface of white matter (Figure S1, *K*). No difference was found among ventral, lateral or dorsal spinal cord regions in both wobbler and healthy mice. No relevant morphological alterations were also observed in Hoechst 33258 positive nuclei at this time of the study (Figure S1, *L*).

A relevant decrease in the number and in the brightness of Hoechst 33258 positive nuclei was revealed in both wobbler and healthy mice sacrificed 28 days after graft. This trend was mainly observed close to the site of administration (Figure S1, *M*) and at the third ventricle (Figure S1, *N*). However, also the other regions of the ventricular system (Figure S1, *O* and *P*) showed a slight decrease of Hoechst 33258-related signal. Figure S1, *Q* shows the presence of Hoechst 33258 positive nuclei in the peripheral region of ventral horns in a wobbler mouse. In this picture the grey matter (light green) is easily distinguishable from the white matter (blue). It is interesting to notice the lack of localization of Hoechst 33258 positive nuclei close to the affected region (lamina VIII and lamina IX).

A further reduction of Hoechst 33258 positive nuclei was observed in mice sacrificed 56 days after hAFC transplantation (Figure S1, *R*-*U*). At the spinal cord level the number of sections showing light-blue fluorescent nuclei was drastically reduced without any particular difference between wobbler and healthy mice. The few remaining nuclei showed a marked loss of brightness but not relevant morphological alterations (Figure S1, *V*).

*Lymphocytes infiltration in choroid plexus of transplanted mice*

To evaluate any eventual local inflammation with recruitment of lymphocytes, immunohistochemistry for CD3, CD4 and CD8 was performed 28 days after ICV administration of PBS or SPIOn-labeled hAFCs. A very low expression of CD3 (Figure S2, *A*), CD4 (Figure S2, *B*) and CD8 (Figure S2, *C*) was observed inside the lateral ventricle of PBS injected mice, with a minimal signal in the brain parenchyma close the ventricles. A comparable situation was retrieved for CD3 (Figure S2, *D*), staining in a wobbler mouse 28 days after SPIOn-labeled hAFC graft. Conversely, a moderate increase of both CD4 (Figure S2, *E*) and CD8 (Figure S2, *F*) immunoreactivity was observed in comparison with PBS-treated mice (Figure S2, *C* and *D*). In both groups CD4 and CD8 positive cells were mainly observed corresponding to choroid plexus.

To support our findings the strong immunoreactivity in sections of spleen is shown as positive control in Figure S2, *G-I*
